# Fracture resistance of simulated immature teeth restored with different modalities

**DOI:** 10.6026/973206300214475

**Published:** 2025-12-15

**Authors:** Aishwarya Sinha Mathur, Shailja Singh, Neetu Rani, Shiv Darshan Rao, Ritu Sharma, Shazia Siddiqui, Ganiga Channaiah Shivakumar

**Affiliations:** 1 Department of Dentistry, Gautam Buddha Chikitsa Mahavidyalaya, Ras Bihari Bose Subharti University, Dehradun, Uttarakhand, India; 2 Department of Conservative Dentistry and Endodontics, Faculty of Dental Sciences, KGMU, Lucknow, Uttar Pradesh, India; 3 Department of Periodontology, Faculty of Dental Sciences, KGMU, Lucknow, Uttar Pradesh, India; 4 Department of Oral & Maxillofacial Surgery, Teerthanker Mahaveer Dental College & Research Centre, Moradabad, Uttar Pradesh, India; 5 Department of Conservative Dentistry and Endodontics, School of Dental Sciences, Sharda University, Greater Noida; 6 Department of Conservative Dentistry and Endodontics, Career Postgraduate Institute of Dental Sciences and Hospital, Lucknow, Uttar Pradesh, India; 7 Department of Oral Medicine and Radiology, People's College of Dental Sciences and Research Centre, People's University, Bhopal, Madhya Pradesh, India

**Keywords:** Immature teeth, mineral trioxide aggregate, biodentine, glass fiber post, fracture resistance, endodontic reinforcement

## Abstract

Immature permanent teeth with thin dentinal walls are highly prone to fracture, creating a major challenge in restorative management. Therefore, it is of interest to evaluate the fracture resistance of simulated immature teeth restored using different reinforcement modalities. Forty extracted single-rooted human canines were standardized, simulated as immature teeth, and allocated into five groups: MTA-Gutta percha, MTA-MTA, MTA-Biodentine, MTA-Glass fiber post, and positive control, followed by fracture testing in a Universal Testing Machine. The MTA-Glass fiber post group demonstrated the highest fracture resistance, while the unfilled control group showed the lowest, with significant differences between the control and most reinforced groups. Overall, MTA and Biodentine provided reinforcement, but the combination of apical MTA and a glass fiber post yielded the greatest improvement in fracture resistance.

## Background:

The preservation of teeth, of which the apical development has been arrested due to certain conditions, such as trauma, is a very important issue in dentistry. Any extraction followed by implant supported crowns or a fixed prosthesis in the earlier years of life may not be feasible treatment options owing to the ongoing craniofacial growth process [1]. The endodontic treatment of young teeth presents numerous challenges for doctors. The contaminated root canal space cannot be sterilized using conventional endodontic techniques with files. Additionally, the filling of the root canal is challenging due to the open apex, which lacks a barrier for the root filling material, thus leading to the encroachment of the material on the periodontal tissues. Furthermore, the roots of these teeth are slender, exhibiting an increased vulnerability to fracture. These limitations diverted interest towards therapies that aimed to induce a calcified barrier in a root with open apex. These interventions are termed as apexification procedures. The material of choice for these procedures is MTA, although newer materials such as Biodentine have also been introduced [2]. Various modalities can be considered for restoration of the root canal space after MTA apexification, namely, gutta percha, Biodentine and MTA itself [3]. Nonetheless, the fracture resistance of such teeth remains unknown despite a successful repair operation. Therefore, it is of interest to assess the fracture resistance of simulated immature teeth repaired using various methods.

## Materials and Methods:

## Sample selection and storage:

Forty newly extracted, single-rooted intact human permanent canines, devoid of decay, prior restorations, fractures, or developmental anomalies, were procured and meticulously debrided of all soft and hard tissue debris utilizing an ultrasonic scaler (DTE D5, Gullin, Woodpecker Medical Instrument Co. Ltd.). The samples were thereafter preserved in a 0.5% Chloramine-T solution (Hi-Media Labs, India) until needed.

## Sample fabrication:

To simulate immature apices for all teeth, the size of the apical foramen was enlarged until a number 5 Peeso reamer could be passed through completely. The coronal section was excised to the middle third using a diamond disc in a straight handpiece (NSK, Eschborn, Germany) with water coolant, replicating an Ellis Class III fracture, while standardizing the overall length of all teeth at 14mm. Precautions were implemented to avert contamination and dehydration of the exposed dentin surface by preserving the samples in Chloramine-T solution till subsequent utilization. The chosen teeth were subsequently affixed in self-curing acrylic resin via Teflon molds, embedding the roots into the resin up to 2mm beneath the cementoenamel junction, with their incisal surfaces oriented upwards. The dentin surfaces of the mounted samples were meticulously cleaned with running water for 20 seconds and then blot-dried to achieve a clean and adequately dry surface. Access openings were created in all samples utilizing a round bur, followed by canal debridement with 3% sodium hypochlorite. Out of the forty specimens, thirty-six were then randomly divided into four groups:

[1] Group 1- MTA (MTA ANGELUS®, Angelus Dental) plug placed of 4mm + radicular space restored by lateral compaction of Gutta percha.

[2] Group 2- MTA plug placed of 4mm + radicular space restored with MTA (MTA ANGELUS®)

[3] Group 3- MTA plug placed of 4mm + radicular space restored with Biodentine (Biodentine™, Septodont)

[4] Group 4- MTA plug placed of 4mm + radicular space restored by placement of glass fiber post (3M™ RelyX™ Fiber Post 3D Glass Fiber Post) by cementation with Paracore () resin luting cement.

[5] The four remaining specimens were kept as positive controls, in which neither an MTA apical plug was placed nor the radicular space was restored, that is, Group 5.

All the samples were then sealed coronally by nanohybrid composite resin. The samples were then subjected to testing for fracture resistance under a Universal Testing Machine (Saumya Technologies, UTM-D2). The peak load for fracture was recorded.

## Results:

The mean values for fracture resistance of all the Groups are shown in Figure 1 (see PDF). The highest mean value for fracture resistance was seen in Group 4, that is, MTA-Glass fiber group; whereas, the lowest mean value for fracture resistance was seen in Group 5, that is, the positive control group. On pair wise comparison of fracture resistance values of each test group with the control group, statistically significant difference was observed in all except Group 1, that is, MTA-Gutta percha group (Table 1 - see PDF). On pair wise comparison of fracture resistance values between the different test groups, statistically significant difference was seen in all except between Group 2 (MTA-MTA) and Group 3 (MTA-Biodentine) (Table 2 - see PDF).

## Discussion:

Apexification and root reinforcement may mitigate the diminished mechanical characteristics associated with the incomplete development of juvenile permanent teeth. MTA has established itself as the benchmark in treatment protocols due to its characteristics, including a high pH (12.5 when set), antimicrobial properties, biocompatibility, low cytotoxicity, effective sealing capability, and the ability to set in the presence of blood and moisture, as well as its demonstrated efficacy in promoting apical hard tissue formation [4]. In relation to the vulnerability of maxillary anterior teeth to trauma, maxillary canines were chosen for the investigation. The coronal segment was not rehabilitated with crowns to eliminate any external reinforcement effects. Nonetheless, any inference drawn by comparison to a clinical scenario cannot be established without the application of crowns; thus, this may represent a limitation of the study. In *in vitro* load testing, tooth is mounted in a resin block, which has limited resiliency as compared to the viable periodontal ligament and alveolar bone. Thus, loading using an *in vitro* experiment may not be representative of the *in vivo* condition [5]. In the present study, the teeth restored with glass fiber posts were actually stronger than all other groups and showed highest fracture resistance under load (P < 0.05). The findings of this study align with those of Schmoldt *et al.*, which demonstrated that fiber posts substantially enhance tooth strength and may reduce the likelihood of fractures [6]. This observation may be associated with a comparable modulus of elasticity between fiber posts and dentin, as well as a more uniform distribution of force along the root [6, 7]. Moreover, fiber posts have demonstrated an enhancement in the flexural characteristics of the core composite encasing the post [8]. The luting of a glass fiber post with dual-cure, glass-reinforced composite resin, Para Core, may have resulted in a monobloc structure [6].

The teeth reinforced with gutta percha showed the least fracture resistance among the filled groups. Williams *et al.* concluded that the cohesive strength and modulus of elasticity of gutta-percha is too low to reinforce the roots of an immature tooth [9], which might be a reason for lowest values of fracture resistance under load in this study as well. In groups where teeth were reinforced with MTA and Biodentine, the fracture resistance values for those filled with MTA exceeded those filled with Biodentine. In a study conducted by Zhabuawala *et al.* it was reported that under optimal oral conditions, the fracture resistance of teeth reinforced with MTA improved over time compared to those reinforced with Biodentine, as MTA promotes the expression of tissue inhibitor metalloproteinase-2 in the dentin matrix and inhibits the degenerative actions of MMP-2 and MMP-14 [10]. Further investigation is required to determine if a similar phenomenon may be found with Biodentine. Sawyer *et al.* asserted that the fracture resistance of roots is unaffected when calcium silicate-based compounds, such as MTA and Biodentine, are utilized as apical plug materials. Nonetheless, the thorough obturation of root canals with these calcium silicate-based compounds may diminish the fracture resistance of teeth [11, 12]. Leiendecker *et al.* similarly concluded that extended exposure of mineralized dentin to calcium silicate cements negatively impacts the integrity of the dentin collagen matrix [13, 14]. Pertaining to the positive control group, that is, the unfilled teeth, they showed the least resistance to fracture under load. This finding establishes the fact that immature pulpless teeth should not be left as such and should undergo treatment which provides reinforcement. Research indicates that in pulpless immature teeth, the abrupt cessation of root dentinogenesis results in a thin root wall characterized by incompletely formed peritubular and intertubular dentin, exhibiting increased tubular density near the cementum. Conversely, when mature teeth are modified to mimic immature teeth, the outer regions of their roots display reduced tubular density and a greater proportion of intertubular dentin [15, 16-17]. Consequently, this suggests that while these teeth may morphologically resemble juvenile teeth, they lack comparable tissue composition or physical features, which may be a hindrance for such studies.

## Conclusion:

The facture resistance of unfilled pulpless immature teeth is the least. Obturation of radicular space with gutta percha after placement of apical plug of MTA increases their fracture resistance but not significantly. Restoration of the radicular space with MTA or Biodentine provides considerable reinforcement and increases the fracture resistance. The greatest resistance to fracture can be achieved by the placement of glass fiber post in the radicular space after apical placement of MTA.

## References

[1] Elnaghy A (2020). Clin Oral Investig..

[2] Aktemur Türker S (2021). J Dent Res Dent Clin Dent Prospects..

[3] Das S (2023). Cureus..

[4] Tuna EB (2011). Dent Traumatol..

[5] Asmussen E (2005). J Prosthet Dent..

[6] Schmoldt SJ (2011). J Endod..

[7] Tay FR, Pashley DH (2007). J Endod..

[8] Hattori M (2010). J Dent Mater..

[9] Williams C (2006). J Endod..

[10] Zhabuawala MS (2016). J Indian Soc Pedod Prev Dent..

[11] Sawyer AN (2012). J Endod..

[12] Leiendecker AP (2012). J Endod..

[13] Topçuoglu HS (2015). Int J Paediatr Dent..

[14] Ali MRW (2019). Acta Biomater Odontol Scand..

[15] Shaheen NA, El-Helbawy NGED (2016). World J Dent..

[16] Mello I (2020). J Endod..

[17] Carvalho CA (2005). Dent Traumatol..

